# Vestibular Injury After Low-Intensity Blast Exposure

**DOI:** 10.3389/fneur.2018.00297

**Published:** 2018-05-14

**Authors:** Steven Lien, J. David Dickman

**Affiliations:** ^1^Department of Neuroscience, Baylor College of Medicine, Houston, TX, United States; ^2^Department of Biosciences, Rice University, Houston, TX, United States; ^3^Department of Psychology, Rice University, Houston, TX, United States

**Keywords:** blast injuries, vestibuloocular reflex, optokinetic response, traumatic brain injury, semicircular canal damage

## Abstract

The increased use of close range explosives has led to a higher incidence of exposure to blast-related head trauma. Exposure to primary blast waves is a significant cause of morbidity and mortality. Active service members and civilians who have experienced blast waves report high rates of vestibular dysfunction, such as vertigo, oscillopsia, imbalance, and dizziness. Accumulating evidence suggests that exposure to blast-wave trauma produces damage to both the peripheral and central vestibular system; similar to previous findings that blast exposure results in damage to auditory receptors. In this study, mice were exposed to a 63 kPa peak blast-wave over pressure and were examined for vestibular receptor damage as well as behavioral assays to identify vestibular dysfunction. We observed perforations to the tympanic membrane in all blast animals. We also observed significant loss of stereocilia on hair cells in the cristae and macule up to 1 month after blast-wave exposure; damage that is likely permanent. Significant reductions in the ability to perform the righting reflex and balance on a rotating rod that lasted several weeks after blast exposure were prominent behavioral effects. We also observed a significant reduction in horizontal vestibuloocular reflex gain and phase lags in the eye movement responses that lasted many weeks following a single blast exposure event. OKN responses were absent immediately following blast exposure, but began to return after several weeks’ recovery. These results show that blast-wave exposure can lead to peripheral vestibular damage (possibly central deficits as well) and provides some insight into causes of vestibular dysfunction in blast-trauma victims.

## Introduction

Blast exposures are unfortunately increasingly becoming a common experience in modern society (1). The incidence of blast exposures to military and civilian populations has increased dramatically in recent years, mainly due to the increased use of improvised explosive devices (IEDs). Hearing and balance disorders, including tympanic membrane (TM) perforations, hearing loss, dizziness, vertigo, gaze instability, postural deficits, and spatial disorientation are among the most common symptoms reported in blast-exposed patients (2–8). Further accumulating evidence suggests that these blast exposures may result in undetected damage to both the vestibular receptors, primary afferents, and central vestibular circuits (9–12). For example, recent studies of returning combat personnel exposed to blast-wave trauma have consistently reported incidents of dizziness and postural instability (5, 13). Surprisingly, examination of the vestibuloocular reflex (VOR) in these patients have resulted in inconsistent observations, which is likely explained by the large variance in the strength of the blast the patients were exposed to; the number of exposures each patient experienced; the orientation of the head to the blast wave; and the type of head protection the patients were wearing, if any (14). Although vestibular effects due to blast-wave exposure have been observed, currently the location and extent of vestibular damage following blast exposure remains unknown. However, self-report questionnaires, clinical measures, and physiologic testing indicate a likely vestibular related etiology, possibly through damage to the peripheral inner ear vestibular receptors and central vestibular nuclei (9, 15–18).

Following blast exposure, there are 4 different mechanisms of damage (19). Primary blast injury is caused by the direct effect of the high overpressure wave on tissue. Secondary blast injury occurs when the blast wave propels shrapnel and debris into the tissue. Tertiary blast injury is when the blast wave propels the individual into a solid object, such as the ground or a wall. Quaternary blast injury refers to all other effects such as post-traumatic stress disorder or burns. Primary blast injury is most commonly seen where there is a change in density, such as tissue-air junctions (20). The inner ear and lungs are especially susceptible to primary blast injury due to their interactions with surrounding air. Blast-wave damage can be affected by both the position and direction facing relative to the blast (21).

We have utilized a blast-wave generator (shock tube) that delivers an air shock waveform which closely mimics that produced by IEDs (22, 23). Recent laboratory work in mouse models using similar blast devices reports evidence of blast-induced trauma on the cochlea and the spiral ganglion, which is in close proximity to the vestibular organs (24). Furthermore, mice exposed to blast waves have been observed to perform poorly in spatial navigation and motor coordination tasks. Further, recent evidence from Koliatsos et al. (25) indicates that blast-wave trauma causes widespread cell death in the brainstem, hippocampus, and cerebellum. However, none of these studies examined either the vestibular peripheral receptors or vestibular eye movement responses so it is unclear how these structures and functions are affected by blast exposure. Identifying the effects of blast-wave exposure on the vestibular system, particularly the areas of injury is likely to enhance our ability to understand and treat the vestibular pathologies that are a common source of blast-exposed patients. The objective of this study was to develop a model that would allow us to study the effects of blast trauma, similar to that of an IED, on vestibular reflexes and the peripheral vestibular system.

## Materials and Methods

### Animals

In this study, male or female C57/Bl6 mice (aged 12–18 weeks) were used for all experiments. Mice were housed in the Comparative Center for Medicine at the Baylor College of Medicine vivarium. All experiments were performed in accordance with the National Institutes of Health guidelines for the humane treatment of animals and were approved by the Institutional Animal Care and Use Committee.

### Blast Chamber

**Figure 1:**
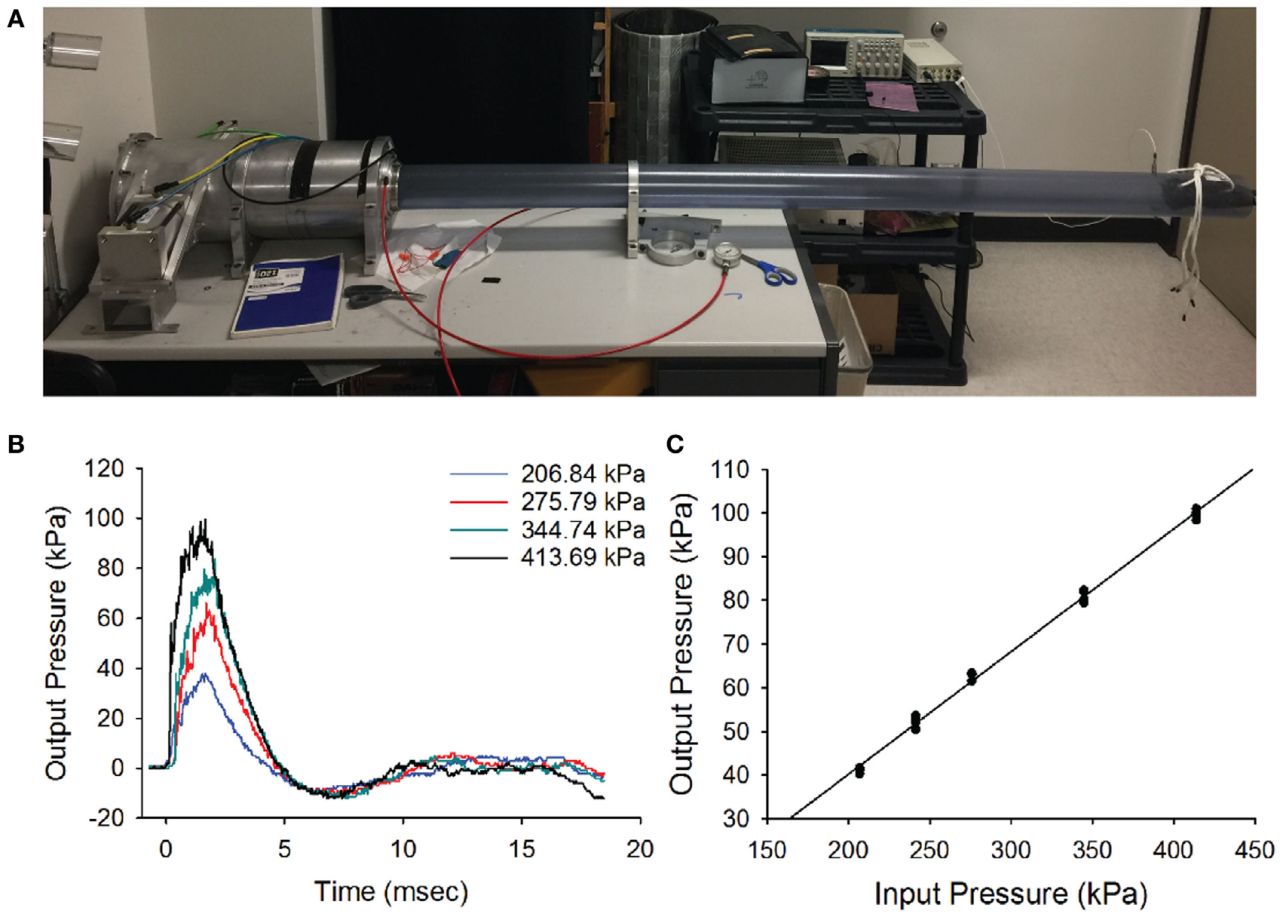
Blast wave. **(A)** The blast-wave device with the mouse holder positioned 8 feet away from the blast chamber. **(B)** Blast-wave profile measured by a pressure transducer mounted directly under the animal’s head as a function of input pressure (colored traces). A rapid onset of the blast-wave pressure (0 ms) is followed by the blast-wave peak at 2–3 ms. The peak is followed by an under pressure wave at 5–7 ms. **(C)** Input/output pressure responses follow a proportional relationship. Each point represents one blast repetition. Black line represents fitted regression.

A previously developed customized air pressure chamber was modified to deliver blast waves to mice (26). The system utilized pressurized air to produce a single compression wave that traveled down the length of a PVC tube (length: 243 cm, diam = 11.5 cm, wall thickness: 0.6 cm) to deliver a shock front to the animal. The outer chamber of the blast generation device was pressurized using a DeWalt D55146 air compressor connected to a pressure modulator (Lumadyne, Inc.). The chamber was driven by a titanium piston that sealed the reservoir from the inner chamber of the blast-wave generator, as shown in Figure 1A.

### Blast Exposure

The blast-wave intensity and temporal profile were measured on an oscilloscope (Tektronix TDS 2014B) for every blast exposure using a high-speed pressure transducer (Model 102B16, PCB piezotronics) that was positioned under the head of the animal (Figure 1B). The chamber was capable of generating peak pressures ranging between 0 and 140 kPa at the position of the head of the mouse.

The blast profiles were first characterized by measuring the stagnation pressure of the airwave at the location of the mouse’s head. Stagnation pressure was measured by positioning the pressure transducer face-on into the blast wave. The stagnation pressure evaluates the sum of both the static pressure within the tube and any dynamic pressure associated with particle motion. As shown in Figure 1B, stagnation pressure measurements demonstrated that our blast-wave profile closely resembled that of an ideal Friedlander function. The blast-wave profile consisted of an overpressure peak at a latency of 1.5–2 ms and a duration of approximately 5 ms, followed by a negative pressure phase that returned to equilibrium. The small perturbations seen in the negative phase originated from reflections of the blast wave in the narrow tube. In an open world situation, these reflected waves would not be as prominent.

We initially tested the response of the blast chamber as an input/output function at five different reservoir pressures, including 206.8 kPa (30 psi), 241.3 kPa (35 psi), 275.79 kPa (40 psi), 344.7 kPa (50 psi), and 413.4 kPa (60 psi). For each of the input pressures, the latency to reach the blast-wave overpressure peak and peak intensity of the wave were recorded. We plotted representative blast-wave pressure profiles versus time at multiple input pressures (Figure 1C). Each point represents one test trial, where multiple tests at the same input pressure were obtained at least 1 week apart. The plot demonstrates that increasing the tank pressure produces higher blast pressures with slightly different positive phase durations. We observed that the blast-wave generator consistently produced blast profiles and peak intensities for multiple repetitions. The minimum input pressure (206.8 kPa) tested produced a blast-wave peak of 41.2 kPa. The maximum input pressure we tested (413.4 kPa) produced a peak intensity of 99.8 kPa (Figure 1C). Increasing the input pressure led to faster peak overpressure latencies and slightly longer overpressure durations. The input vs output pressure peak was linearly correlated (*R*^2^ = 0.99, Figure 1C).

To deliver the blast to the animal, mice were initially anesthetized with a cocktail solution of ketamine (100 mg/kg) and xylazine (10 mg/kg xylazine) before being placed into the chamber. The animal was secured at the end of the blast tube with the head directly facing the impact of the blast wave. To isolate the blast exposure to the animal’s head, the body was secured in a sheath of fiberglass mesh and foam, then stabilized with a stiff bar and straps. We left the front of the sheathe open to fully expose the head of the mouse to the impact of the blast wave. Mice were placed front facing the blast-wave overpressure. Although several blast intensities were initially tested (see Figure 1), we used an intensity of 63 kPa, where a mortality rate <10% and consistent vestibular dysfunction was produced. All animals were exposed to only a single blast wave.

### Rotarod

We measured postural performance on a rotarod device according to the protocol reported by Baalman et al. (26). Before rotarod testing, animals were individually handled through three consecutive days for at least 15 min each. The mice were allowed to habituate in the behavior room for 1 h the day before the rotarod testing was performed. Animals were then trained to perform the rotarod task for 4 days before assessment. On the first day of training, mice were required to maintain balance on a non-moving rod for 20 s and then locomote on the rotating rod at 5 rpm for 90 s. On days 2–4, the rod constantly accelerated from 4 to 40 rpm over a 5 min period to collect normative baseline responses. Animals were then exposed to a single blast-wave and tested on days 1, 7, and 14 after blast-wave exposure. Each animal was tested on three separate trials per day with a 10 min break between each trial. The latency to fall from the rod was averaged for each separate trial.

### Righting Reflex

Righting reflex was tested 1, 7, 14, and 28 days after blast-wave exposure as a measure of postural correction (25). Animals were placed in the supine position, and the time that it took for the animal to correct itself into the prone position was measured.

### Vestibuloocular Reflex

To measure the VOR, a head post was surgically implanted to the skull of each animal. All surgical procedures were performed using 2% isoflurane for anesthesia. Once anesthetized, the scalp was incised, and four stainless steel screws were implanted in the skull around the base of the head post then sealed with dental acrylic. Care was taken to position the head mount so that the plane of the horizontal semicircular canals would be coplanar to an earth horizontal plane. The animal was then removed from the operating table, allowed to recover from the anesthesia, and returned to its home cage.

An infrared eye tracking system (ISCAN ETL-200) was used to record eye movements. A distributed illumination system consisting of three infrared emitters was used to increase luminance values at the edge of the image. Two of the emitters were to the left and right of the axis of the camera. The third emitter was placed directly on top of the camera facing the mouse’s eye. This third emitter was used to produce a reference corneal reflection. Each animal’s eye movements were calibrated to the experimental setup using methods similar to those reported previously (27–30). Briefly, animals were anesthetized with 100 mg/kg ketamine and 10 mg/kg xylazine. The camera was rotated around the center position of the pupil ±10°. The radius of the pupil (Rp) was then calculated by taking the difference of the two extreme positions and dividing it by the amount of rotation. The angular position of the eye *E* was calculated using the Rp value,

(1) $$\text{Rp}=(({\text{Cr}}_{+\text{1}0}-{\text{P}}_{+\text{1}0})-({\text{Cr}}_{-\text{1}0}-{\text{P}}_{-\text{1}0}))/(\text{2}0\pi /\text{18}0),$$

(2) E={(Cr−P)/Rp}.

The Rp was calculated for various pupil sizes by altering the luminance levels within the recording room. A linear fit of the Rp values was taken for each individual mouse. Following calibration, mice were acclimated to the motion system for 3 days before any measurements being taken. The animal was placed head fixed using the implanted head stud into a body restraining carrier that was fixed to the center of the rotational motion system. On the first day, mice were awake and restrained in the dark for 30 min. For the following days, mice were acclimated in the holder for 1 h before being placed back in their home cages. For VOR measurements, mice underwent symmetrical yaw rotation at frequencies, including 0.5, 1.0, and 2.0 Hz at a peak velocity of 20°/s. Blast-exposed animals were tested at 1, 7, 14, and 28 days post blast-wave exposure. Matched control mice were tested at identical time points following placement in the blast tube, but receiving no blast exposure. The animals were rotated for five trials of 10 cycles at each frequency tested in both the light and dark conditions.

Eye movement gains and phase for each frequency were calculated by averaging the responses across the five trials. First, eye movements were converted from pixels into degrees (28) then desaccaded using a low pass filter of 20 Hz. Desaccaded responses were then further examined by eye, and any remaining saccadic segments were manually excluded if necessary. Eye position was converted to velocity by taking the derivative of the position data. A least squares sine fit was performed on both the head velocity and eye velocity traces. Gain was calculated as the ratio of eye velocity to head velocity. Phase was calculated as the temporal difference between peak eye movement velocity and peak head velocity; positive values indicated a phase lag and negative values indicate a phase lead.

### Optokinetic Nystagmus

Mice were head fixed to the motion system as described before. A dome was mounted in front of the mouse that contained a spherical planetarium with randomly placed holes and an internal light source. The light generated a random pattern of dots of three visual degrees in size and produced a radial optic flow stimulus of moving dots at a speed of 5°/s. The mice were exposed to 30 s of darkness followed by 60 s of optokinetic stimulation, followed by 30 s of darkness. Eye position was converted into eye velocity the same way as described for the VOR data. The number of nystagmus beats was counted and slow phase segments isolated. A least squares linear fit was taken of the slow phase data, and OKN gain was calculated as the ratio of the slow phase linear slope to the light rotation speed.

### TM Perforation

We measured size of the TM and any perforations when present, in all mice prepared for anatomical investigations immediately (1 h) after stimulus exposure (*n* = 5 for each exposure group). To determine the size of the TM, the entire area of the TM was first visualized through dissection, and then photographed using a dissection microscope (Olympus ZX12). From the photographs, the outline of the TM was then mapped and the area determined using a planimeter tool in ImageJ. Next, the size of any perforation observed in each animal was measured. The area outlined by the black dashed line indicates the extent of the TM perforation in one blast-exposed animal. Perforation area was divided by the total area for each TM and expressed as a ratio for normalization.

### Antibodies

Antibodies were obtained from Millipore Bioscience Research Reagents and Invitrogen. Phalloidin was chosen as an F-actin stain which we used to label the stereocilia. Calretinin was obtained from Millipore biosciences and was used to label most hair cells and calyx afferents. The primary calretinin antibody was derived from rabbit, and secondary Alexa dye-labeled antibodies were derived from goat.

### Immunohistochemistry

At the end of appropriate post-stimulus survival time, each animal (both control and blast exposed) was deeply anesthetized with sodium pentobarbital (80 mg/kg), and then perfused transcardially with saline, followed by a 4% paraformaldehyde, 5% sucrose, 0.l M phosphate-buffered saline (PBS) solution at pH 7.4. The inner ears were removed and vestibular epithelia dissected out in PBS. Otoconia were removed with gentle agitation (31, 32). Background fluorescence was reduced by incubating the organs in a 1% aqueous solution of sodium borohydride for 10 min. Next, the organs were washed 3× 10 min in PBS. Tissues were then permeabilized with 4% Triton X-100 in PBS for 1 h at room temperature (RT). Tissues were incubated in a blocking solution (0.5% Triton X-100, 0.5% fish gelatin, and 1% bovine serum albumin) in PBS for 1 h at RT. Organs were then incubated in a mixture of blocking solution and calretinin (1:200) for 72 h at 8°C. The organs were washed 5× 10 min each at RT and incubated in a mixture of goat anti-rabbit antibody conjugated to an Alexa Fluor 594 fluorophore (1:400) and phalloidin conjugated to a 488 fluorophore (1:200). Tissues were then rinsed with PBS and mounted on slides in vectashield.

### Stereocilia Quantification

Slides were examined on an Axio Imager 2 (Zeiss) and Nikon A1-Rs microscopes. Final image processing and labeling were done with Adobe Photoshop. Stereocilia were quantified by counting the number of actin bundles that were visible in the central epithelium of the cristae using a 60 µm × 100 µm rectangular counting frame (6,000 µm^2^) that was placed over the central epithelium of the cristae.

Utricle stereocilia bundle counts were performed using three methods. First, the number of stereocilia bundles was counted across the entire surface of the macula in both control and blast-exposed animals. Second, a 100 µm × 300 µm window was placed over the central macula zone. All observable bundles were quantified. Second, a larger surface area of the macule was quantified by generating 15 randomized 50 µm × 50 µm windows over the sensory epithelium of the utricle. The counts within each window were summed across all 15 counting regions of the utricle. We then computed the percent area of the sensory epithelium that was quantified per utricle (2,500 × 15)/(sensory area of each individual utricle). The summed counts of stereocilia bundle were then multiplied by a correction factor (100/percent of sensory epithelium sampled) to obtain estimates of the total number of stereocilia bundles per utricle.

## Results

### Rotarod and Righting Reflex

**Figure 2:**
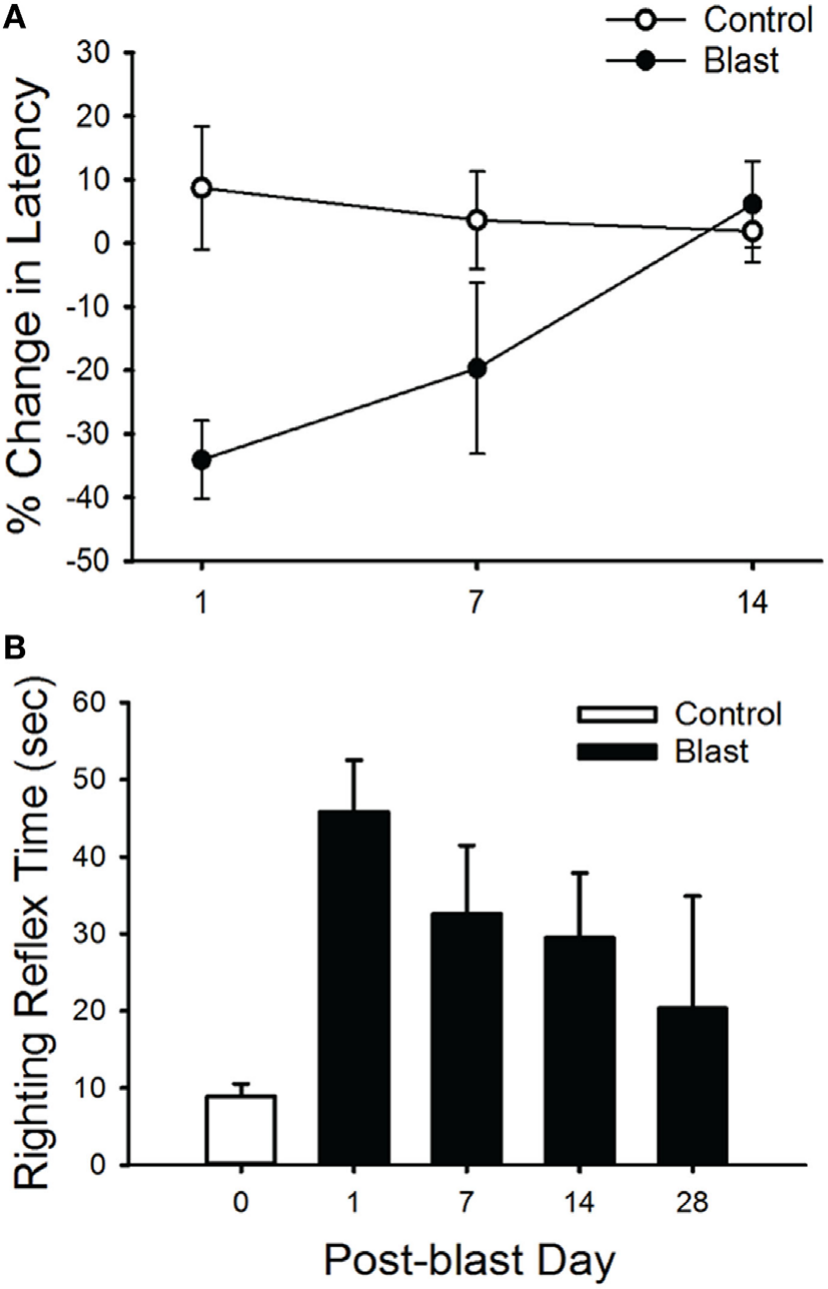
Rotarod and righting reflex responses. **(A)** Animals exposed to a single blast wave (63 kPa) had a significant performance decrease in their mean latency to fall in the rotarod task at 1 and 7 days post-blast exposure (*p* < 0.05). However, there was no significant difference between groups at 14 days post-blast exposure. **(B)** Mean righting reflex responses (time) to control (0 day, white) and post-blast exposed (black) animals, at 1, 7, 14, and 28 days post-exposure. Error bars = SE.

Mice exposed to a single 63 kPa blast wave showed physical impairment on rotarod testing 1 day after exposure, where a 34.1% decrease in rotarod performance was observed as compared with their pre-blast values (Figure 2A, ANOVA *p* < 0.05, *n* = 5 for each group). However, the impaired performance quickly recovered at 14 days after blast-wave exposure, where there was only a marginal difference between the two groups (*p* = 0.064).

In addition, we observed a difference in the righting reflex testing the day after blast-wave exposure (Figure 2B) where mice took significantly more time to right themselves as compared with pre-blast measures (ANOVA *p* < 0.05, *n* = 5 for each group). Before blast-wave exposure, mice exhibited a mean 8.9 s (*n* = 4) to right themselves from a supine position, and on day 1 post-blast, these same mice took 45.8 s to right themselves. The righting reflex deficit persisted through 14 days post-blast exposure. However, by day 28 post-blast, the reflex time was not significantly different from control (ANOVA, *p* > 0.05). Together, these results suggest that exposure to a single blast wave of 63 kPa was sufficient to produce balance deficits in these animals, and the deficit was more pronounced for the righting reflex than rotarod response.

### Vestibuloocular Reflex

**Figure 3:**
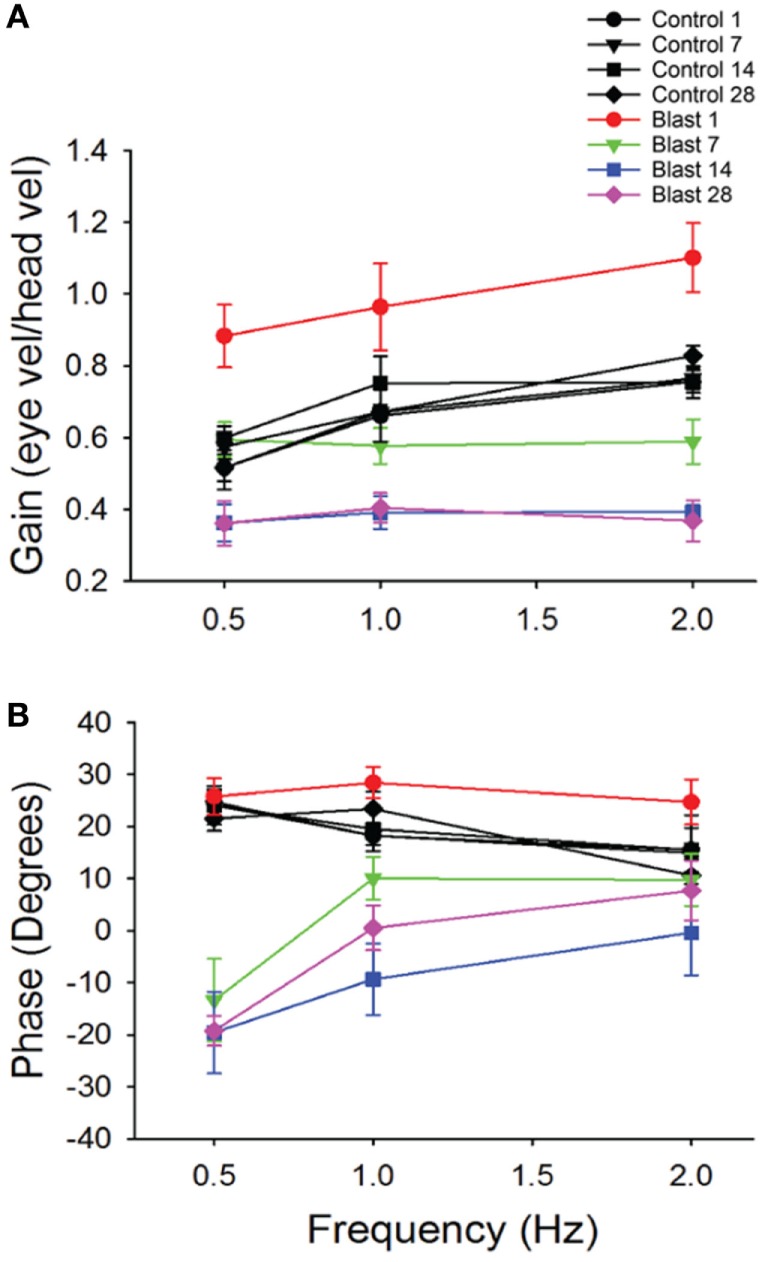
Vestibuloocular reflex (VOR) responses. Mean horizontal VOR responses for control (black, *n* = 10) and blast (colors, *n* = 10) animals on days 1, 7, 14, and 28 post-blast exposure as a function of rotation frequency. Mean horizontal vestibuloocular reflex gain **(A)** and phase **(B)** values for 0.5, 1.0, and 2.0 Hz (20°/s peak velocity). Error bars = SE.

To further investigate possible vestibular physiological effects from blast-wave exposure the horizontal vestibuloocular reflex (hVOR) was measured using sinusoidal rotations in the horizontal head plane. Each mouse was tested on days 1, 7, 14, and 28 after exposure to a single peak 63 kPa blast wave (*n* = 10). As shown in Figure 3, litter-mate control mice (*n* = 10) were tested on the same four post-exposure (no blast—sham) days and exhibited a stable VOR gain with no significant difference observed across testing days (ANOVA *p* > 0.05). Control mice had the lowest gains at a frequency of 0.5 Hz (mean = 0.52 ± 0.04) and the gain increased as the rotation frequency increased (Table 1). Following blast exposure, there was a significant, hypermetric increase in gain above normal values at day 1 post-blast exposure (Figure 3A), with gain increases of 0.36, 0.30, and 0.34 at 0.5, 1.0, and 2.0 Hz frequencies, respectively (Table 1). However, hypermetric responses were not observed across all days. In fact; mice tested on days 7, 14, and 28 exhibited significant decreases in VOR gain across all the frequencies tested (Table 1). Specifically, at 0.5 and 1.0 Hz, day 7 animals exhibited mean gains of 0.52 ± 0.06 and 0.66 ± 0.07, respectively, that were not different from normal (*p* > 0.05). However, day 7 post-blast mice did exhibit a lower gain at 2.0 Hz (*p* > 0.05). For survival post-blast days 14 and 28, all mice exhibited lower than normal VOR gains at all frequencies (Figure 3A; Table 1). In terms of VOR phase, in normal animals there was a phase lead of between 15° and 25°, depending upon rotation frequency (Figure 3B; Table 1). There was no difference in the phase responses from normal values at any frequency for the day 1 post-blast animals (Figure 3B). However, for the 7-, 14-, and 28-day post-blast responses, phase values were significantly different, with values actually lagging the peak rotational velocity by nearly 15° at the lowest frequency of 0.5 Hz tested (Figure 3B; Table 1). As frequency increased, the phase lags lessened for each of the survival post-blast days and at 2.0 Hz were not significantly different from normal (Figure 3B; Table 1).

**Table 1:** Mean horizontal vestibuloocular reflex gain and phase values for control (sham) and post-blast animals.

Animal group (n = 10)	Days post-blast	Frequency (Hz)	Gain	Gain SD	Phase	Phase SD
Sham	1	0.5	0.52	0.04	24.7	2.5
	1	1	0.66	0.07	18.2	1.8
	1	2	0.76	0.03	15.6	6.6
	7	0.5	0.56	0.04	24.1	2.9
	7	1	0.67	0.02	18.2	1.8
	7	2	0.77	0.02	14.9	1.5
	14	0.5	0.60	0.03	24.1	3.7
	14	1	0.75	0.07	19.5	4.2
	14	2	0.75	0.04	15.5	4.2
	28	0.5	0.52	0.06	21.5	2.3
	28	1	0.67	0.01	23.4	3.4
	28	2	0.83	0.03	10.6	3.2

Blast	1	0.5	0.88	0.09	25.8	3.5
	1	1	0.96	0.12	28.4	2.9
	1	2	1.10	0.10	24.7	4.3
	7	0.5	0.60	0.05	13.4	8.0
	7	1	0.58	0.05	10.0	4.1
	7	2	0.59	0.06	9.7	5.0
	14	0.5	0.36	0.05	19.6	7.9
	14	1	0.39	0.05	9.4	6.9
	14	2	0.39	0.03	0.4	8.2
	28	0.5	0.36	0.06	19.3	2.9
	28	1	0.40	0.04	0.5	4.3
	28	2	0.37	0.06	7.7	5.8

### OKN Responses

**Figure 4:**
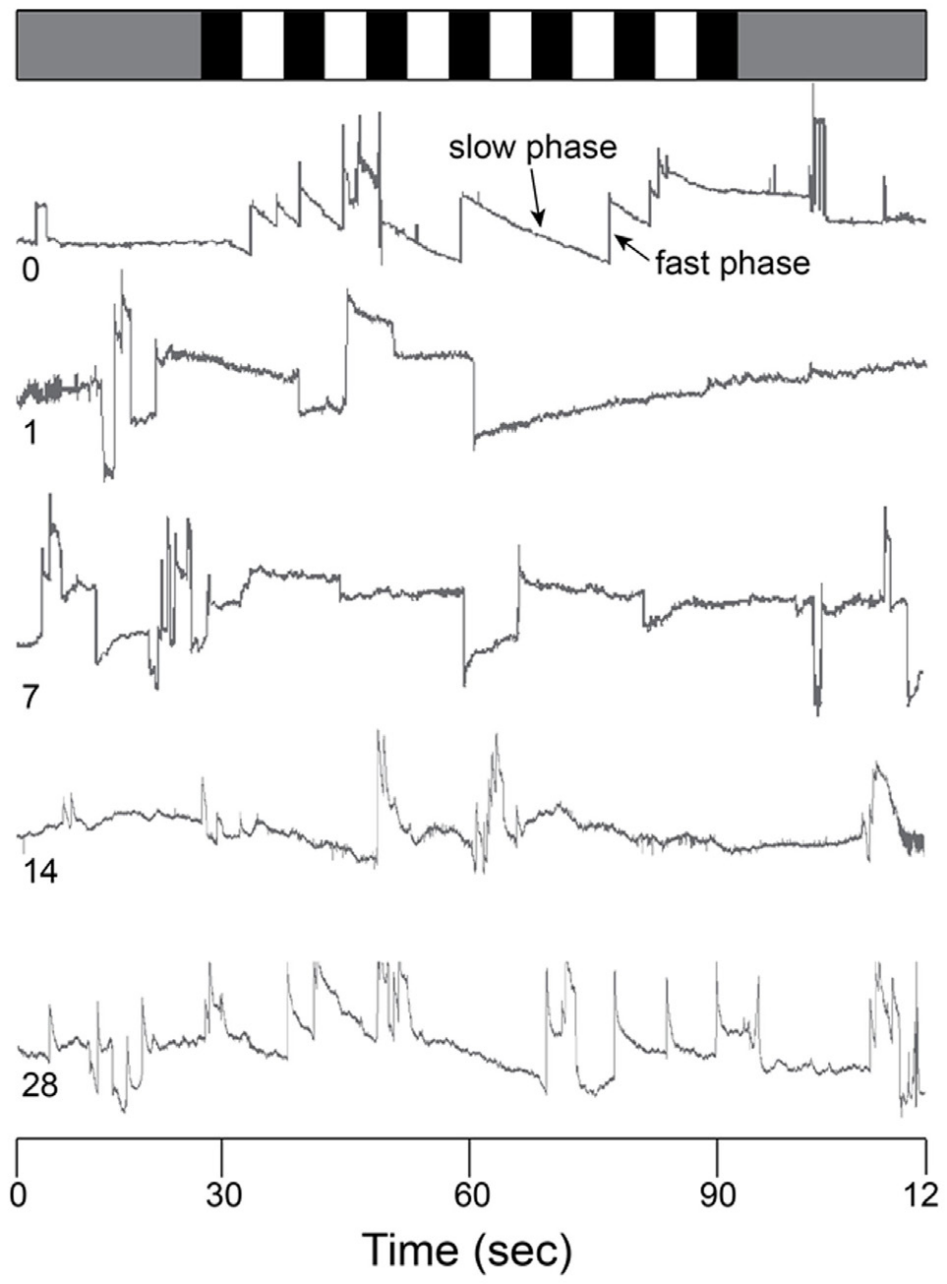
Raw OKN response. Eye position traces for 0 (control), 1, 7, 14, and 28 days post-blast exposure for one animal as a function of time (in s). Gray bar (top) indicates no motion stimulus. Alternating black and white bars indicate onset/offset of optokinetic stimulus. Slow phase and fast phase eye movements are indicated in the day 0 trace (arrows).

**Figure 5:**
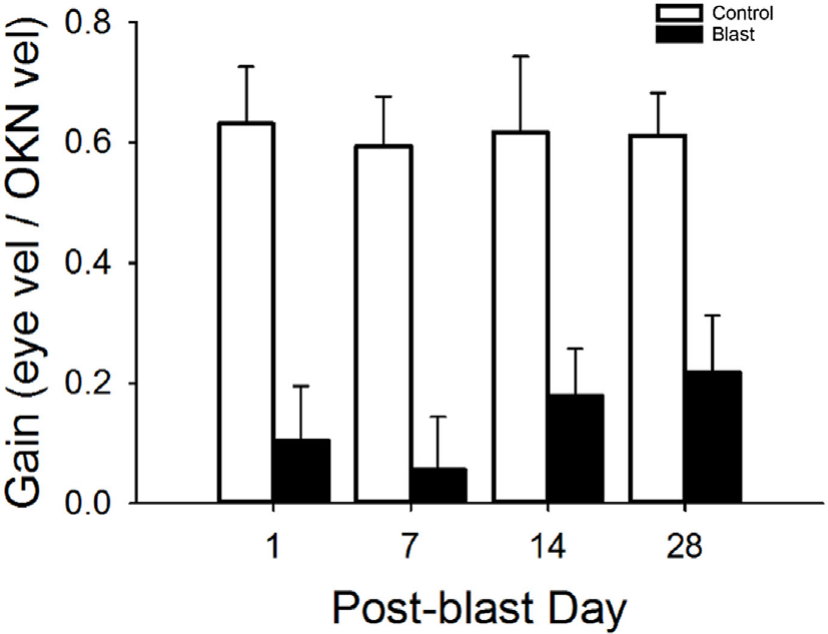
Mean OKN responses. Mean OKN responses for control and blast animals as a function of post-condition day exposure (control = white, black = blast). Error bars = SE.

The optokinetic reflex was examined before and for 28 days following blast exposure in five mice. Before blast (control), the rotating OKN stimulus elicited a series of slow phase/fast phase eye movements in a nystagmic pattern at low stimulus velocities, as shown in Figure 4 for one representative animal response. Before blast, the mean slow phase velocity gain for a 3°/s OKN stimulus velocity was 0.63 ± 0.101 deg/sec per deg/sec (Table 2). On day 1 following blast exposure, we observed a significant reduction in OKN gain, as shown in Figure 5. In fact, on day 1 post-blast, there was no quantifiable slow phase eye velocity that could be observed (Figures 4 and 5). A similar lack of OKN response was observed for most animals on days 1–14. However, a few nystagmus beats were observable in a few mice during this recovery phase and at day 7 post-blast, the mean OKN gain value for the few slow phase episodes present was 0.08 ± 0.09 deg/sec per deg/sec (Table 2). These reductions in OKN response continued through day 14 post-blast exposure and were significantly different from normal (Table 2). Even though, there appeared to be a few slow phase episodes indicating a small degree of recovery by day 14 post-blast; the eye position response resembled an exponential decay function during the slow phase (Figures 4 and 5). This exponential decay pattern continued to be observed at day 28 post-blast exposure, but the number of slow phase episodes was becoming more notable (Figures 4 and 5). The mean gain at 28 days recovery was 0.217 deg/sec per deg/sec (Table 2). The presence of some nystagmus was beginning to return during OKN stimulation in the 28-day blast animals.

**Table 2:** Mean OKN gain values for control and post-blast animals.

Animal group (n = 5)	Days post-blast	Gain	Gain SD
Sham	1	0.63	0.09
	7	0.59	0.08
	14	0.62	0.13
	28	0.61	0.07

Blast	1	0.11	0.09
	7	0.06	0.09
	14	0.18	0.08
	28	0.22	0.09

### TM Perforation

**Figure 6:**
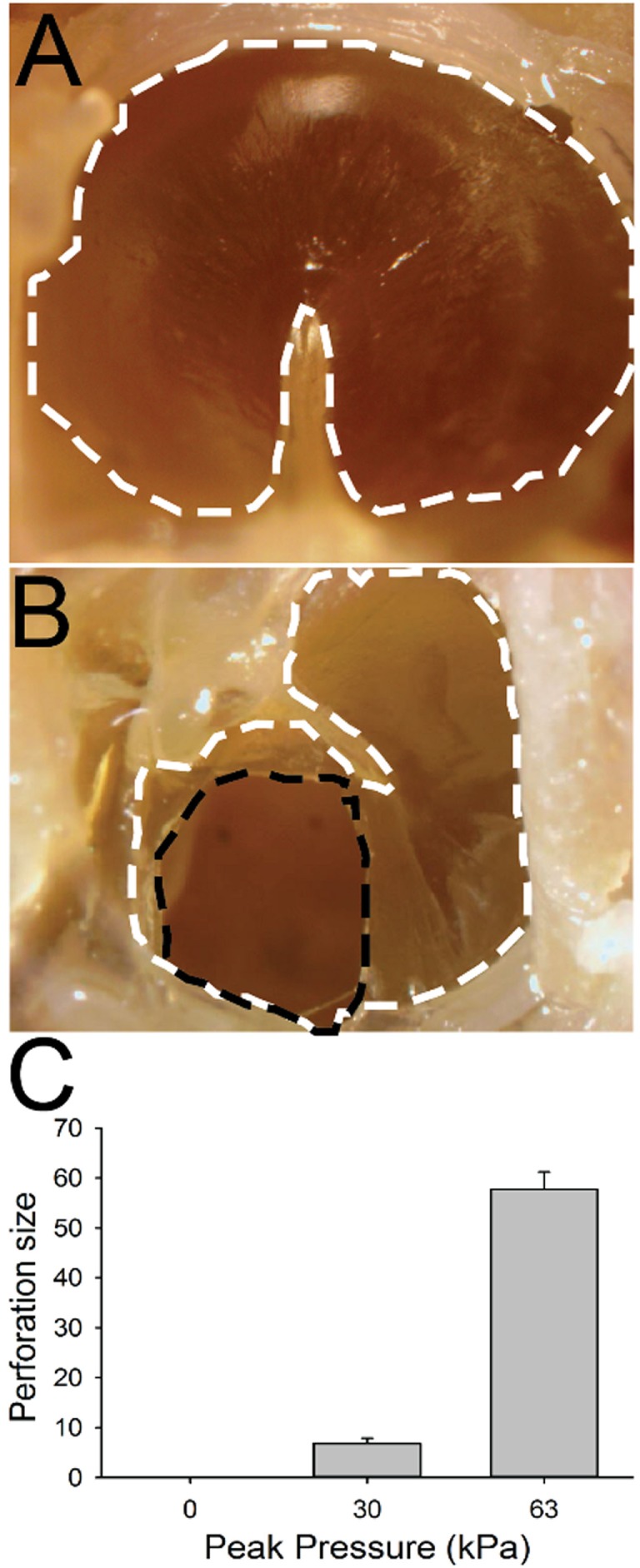
Tympanic membrane (TM) perforation. **(A)** Control animal. The TM is outlined with a white dashed line. **(B)** TM 1 h after 63 kPa blast-wave exposure. The dotted black line indicates blast-wave damage region. **(C)** Perforation size (normalized percent of total area) in blast vs control animals as a function of blast intensity. Scale bar = 1 mm.

In control animals, we did not observe any perforations following sham blast exposure (Figures 6A,C; *n* = 5). Damage to the TM was slightly larger (mean size of 5.9 ± 1.7% of the total surface area, *n* = 5) in mice exposed to a 30 kPa blast, with small but significant perforations observed in all animals (Figure 6C, *p* > 0.05). Mice exposed to a 63 kPa blast wave had significantly larger TM perforations (Figures 6B,C; *p* < 0.05). In these animals, a mean of 57.8 ± 3.5% (*n* = 5) of the total surface area was perforated (Figure 6C).

### Stereocilia Bundle Loss

**Figure 7:**
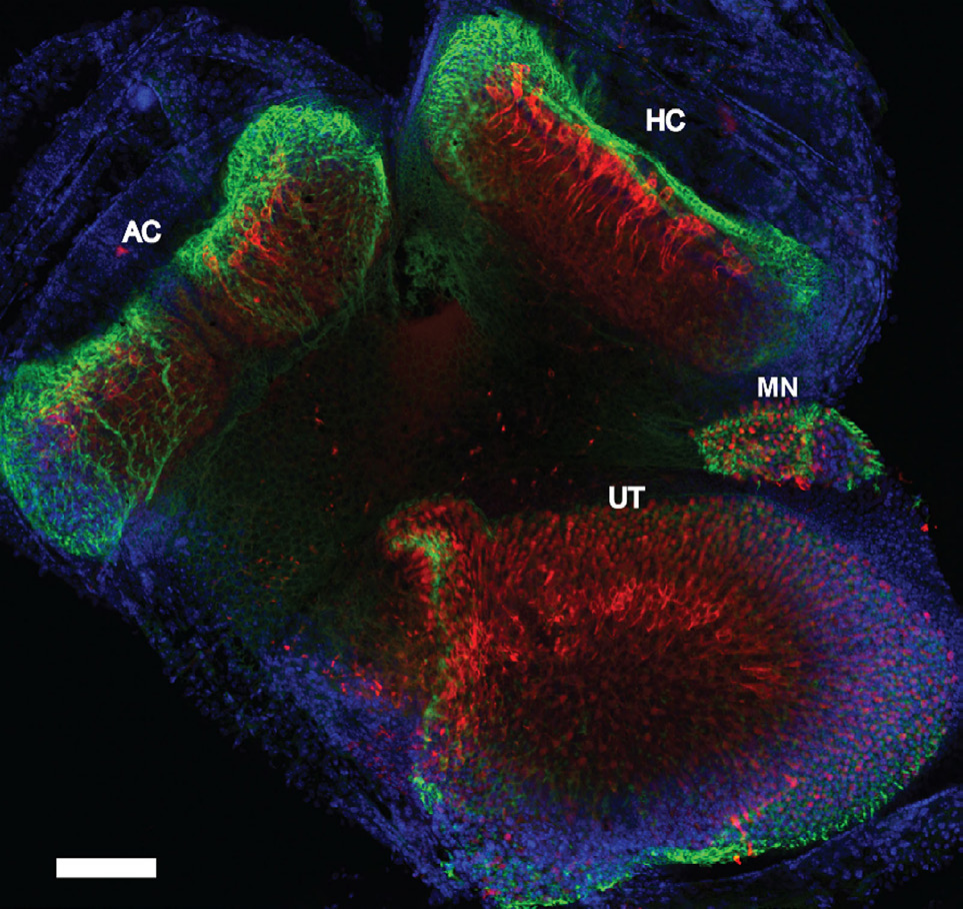
Whole mount immunofluorescence of the vestibular receptor organs. Vestibular receptor organs in a control mouse were labeled with phalloidin (green), calretinin (red), and DAPI (blue). Horizontal semicircular cristae (HC), anterior semicircular cristae (AC), utricle (UT), and macula neglecta (MN). Scale bar = 200 µm.

**Figure 8:**
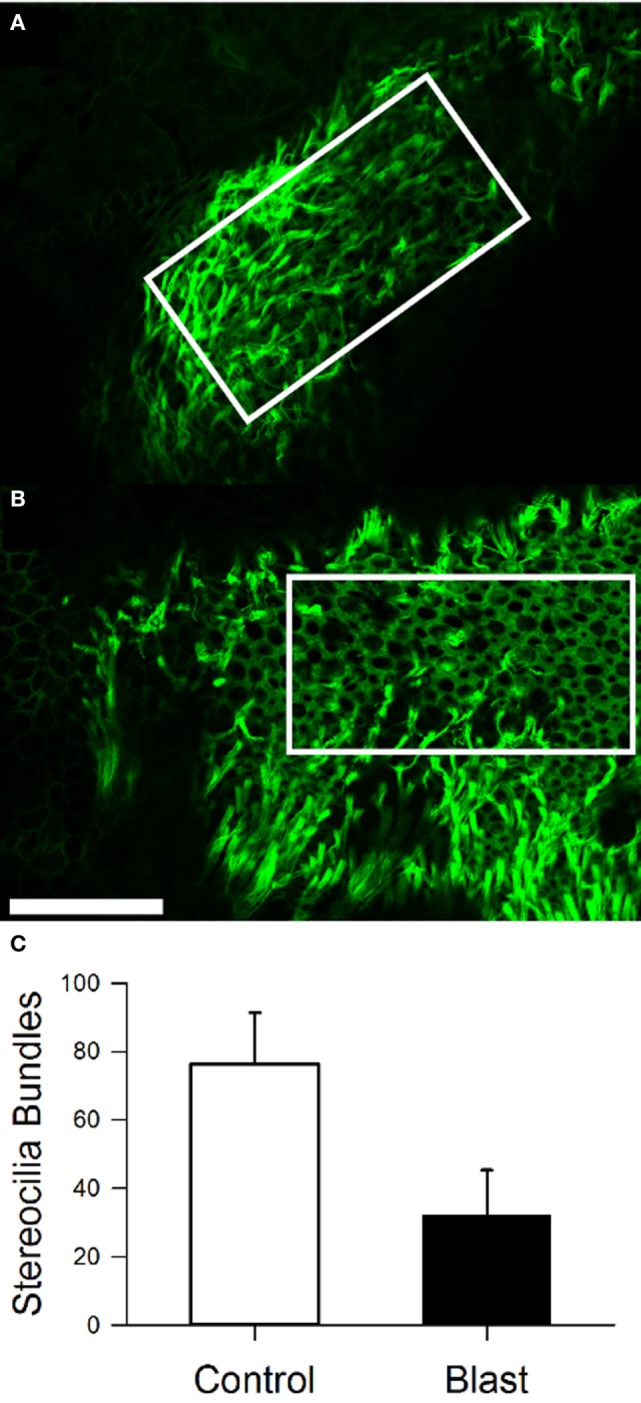
Stereocilia bundle loss in the horizontal cristae observed 28 days after blast-wave exposure. Confocal images of the cristae showing stereocilia (phalloidin, green) in control **(A)** and blast **(B)** animals 28 days after sham or blast exposure. Rectangle indicates 100 µm × 300 µm counting frame over the central zone of the cristae. **(C)** Mean bundle counts for control (white) and blast (black) animals. Error bars = SE. Scale bars: 50 µm.

We performed immunostaining on the vestibular receptor epithelia. For quantification of the number of stereocilia bundles, we used phalloidin, a peptide that binds F-actin with high selectivity, and calretinin, selective for type I hair cells. As shown in Figure 7, for one control mouse, a triple label of the whole mount tissue that contained the anterior semicircular cristae, horizontal semicircular cristae, utricular macula, and macula neglecta was often utilized, and then each individual organ was further dissected for quantification. As shown in Figure 8, hair cell loss was quantified by counting the number of observed stereocilia bundles in a comparable region of interest for both control and blast-exposed animals. First, the numbers of stereocilia were counted (Photoshop) in the central zone of the horizontal semicircular cristae using a 60 µm × 100 µm region counting frame placed over the central zone. Due to the curved nature of the cristae, we did not attempt to quantify the peripheral zones in these specimens. In control mice (Figure 8A), we observed a mean of 75.2 ± 5.4 (*n* = 10) stereocilia bundle counts in the 6,000 µm^2^ area. However, significant stereocilia bundle loss occurred (Figure 8B) in the HC of blast mice 1 month after blast-wave exposure was observed, with a mean of 31.1 ± 2.9 (*n* = 10) stereocilia bundles per counting frame (Figure 8C). Our data show that one moderate level blast-wave exposure is sufficient to cause significant hair cell loss in the horizontal semicircular cristae.

**Figure 9:**
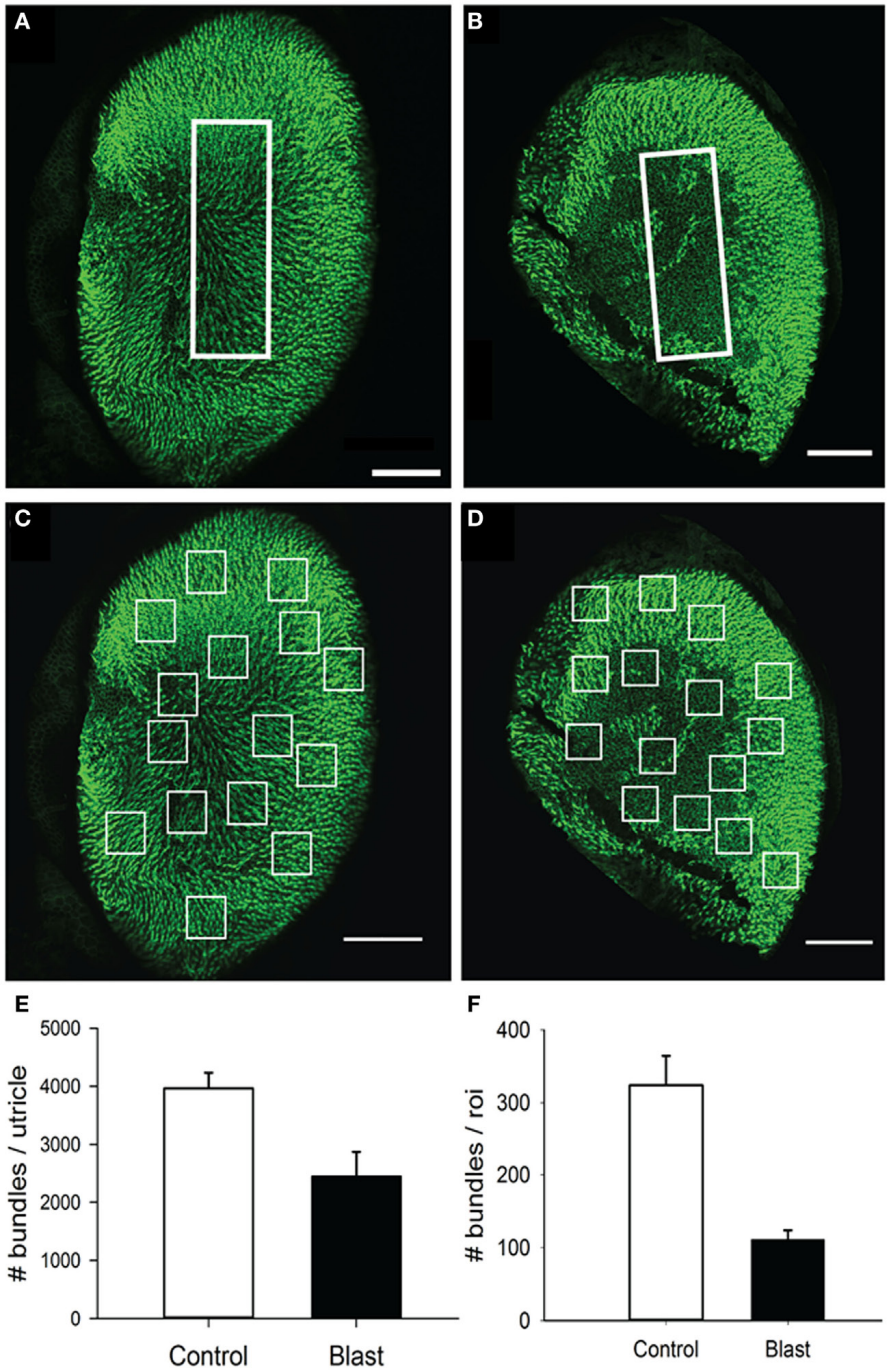
Stereocilia bundles in the utricle. Images of the utricle from in control **(A,C)** and blast **(B,D)** animals at 28 days after sham or blast exposure. Stereocilia bundles were stained with phalloidin (green). **(A,B)** Stereocilia counts were obtained from a central zone counting frame (60 µm × 100 µm, white box). **(C,D)** 15 Random placed counting frames (50 µm × 50 µm) were generated to obtain estimates of the total number of stereocilia bundles per utricle in both control and blast animals. **(E)** The mean number of stereocilia bundles for the central zone counting frame in control (white) or blast (black). **(F)** Mean stereocilia bundle counts averaged across the 15 random counting frames for control (white) or blast (black) animals. Scale bars: 100 µm.

In the utricle, because the receptor epithelium is essential planar, we first counted every stereocilia bundle visible across the entire macular surface, with an average 3,965.6 ± 268.6 (*n* = 5) stereocilia bundles per macula being observed in control animals (Figure 9A). In contrast, animals exposed to the 63 kPa blast wave exhibited significant less stereocilia counts, with an average 2,454.2 ± 421.2 (*n* = 5) stereocilia bundles per utricle (Figure 9E). To further refine the description of the most damaged regions of the macule, we consistently observed that the central zone in blast animals was largely devoid of stereocilia bundles (Figure 9B). Control mice had on average 324 ± 40.3 (*n* = 5) stereocilia bundles in the 100 µm × 300 µm counting frame (30,000 µm^2^) placed in the macula central zone, while the blast mice exhibited a significantly decreased number of stereocilia bundles (111 ± 12.9, *p* < 0.05, *n* = 5). As a final measure of stereocilia loss in the utricular macula, 15 smaller counting frames (50 m × 50 m) were randomly positioned over the surface in each macula (Photoshop) and the number of stereocilia bundles within each frame quantified (Figures 9C,D). The number of stereocilia bundles observed was then averaged across all of the 15 frames for each macula. As shown in Figure 9F, a mean of 324 ± 40.3 (*n* = 5) bundles for the control mice was observed compared with a significantly lower number of 135 ± 40.1 (*n* = 5) bundles in the blast animals (Figure 9F, *p* < 0.05).

## Discussion

Here, we show that moderate whole body blast exposure can induce significant damage to vestibular receptor structures and impair vestibular response behavior. We examined several key commonly assessed vestibular response behaviors including the righting reflex, the rotarod test, the hVOR, and the OKN. We observed impairment in all of these response behaviors immediately after the initial blast exposure, with some improvement over time. We limited our anatomical investigation to an inspection of the TM and vestibular receptor organs. For the vestibular receptors, we concentrated upon damage to the stereocilia of the receptor cells in the horizontal semicircular canal and utricular macula. We observed significant damage to the stereocilia, being eliminated in large sections primarily of the central zones of these receptor organs following a single 63 kPa blast exposure. Examination of the finer structure of underlying hair cells and innervating afferent fibers is currently being addressed in a separate report.

To accurately assess vestibular damage due to blast exposure, it was important to utilize a reproducible blast generator. Our results show that the blast chamber used for these studies was able to reliably produce shock waves with a Friedlander profile similar to those exhibited by commonly encountered IEDs (23). Our model seeks to replicate a shock front that would be experienced during an open field blast exposure. An open field blast wave exposes the animal to a shock front that has been measured to contain peak rise time latencies ranging between 0.15 and 0.3 ms (22, 33, 34). The blast wave produced by our generator consisted of a slightly longer latency to peak (1–2 ms) but was of similar duration and matched intensity to IED explosions in the real world. The generated shock wave amplitudes were proportional to the input pressure utilized and were consistently reproducible over many weeks time. In a pilot study, we initially exposed animals to four different levels of blast intensity, ranging from 30 to 90 kPa. We observed that the intensity of 63 kPa resulted in less than 10% mortality rate and reliably produced vestibular deficits in all animals. Lower intensities had higher survival rates, but inconsistent vestibular insults. Higher intensities (>70 kPa) resulted in greater than 25% mortality rates, largely due to lung damage and were thus not utilized here. In the human literature, it has been difficult to assess the level of blast exposure on an individual basis due to inconsistent parameters, such as the proximity to blast source, intensity of the shock wave, orientation to the shock wave, and the number of exposures encountered (14). These factors likely explain the large variance in findings in patient vestibular measures following blast exposure (35, 36).

We examined the TM in every animal for signs of perforation after exposure to a 30 or 63 kPa blast wave. There was significant damage to the TM at the 63 kPa peak overpressure, in all animals examined. This was not true for the lower 30 kPa blast level animals, where only partial perforations were observed in some animals. TM perforations have been reported to be the most common blast-related injury in combat personnel (37, 38) and terrorism attacks (39). Near universal TM perforations have been observed in controlled laboratory studies in mice and chinchillas using similar blast methodologies and the larger of the intensities to those incorporated here (24, 40). Cho et al. (24) reported spontaneous closure of the perforations over time (approximately 1 month). Although a perforation in the TM is not always associated with damage to the inner ear, it does indicate that enough force has been exerted to possibly produce systemic damage to the vestibular receptors.

In this study, examination of the peripheral vestibular system receptors was limited to assessing the integrity of the stereocilia in the horizontal semicircular canal crista and utricular macula. There was significant stereocilia loss observed in the central zones of both receptor organs, while the peripheral regions were typically left undamaged. We observed that following a single blast exposure, the number of stereocilia in the central regions were reduced to about half of the normal number. Most of the stereocilia damage observed consisted of a complete loss of all cilia on each affected hair cell. Damage remained throughout the 28 days post-blast exposure period, suggesting that little stereocilia repair during this time frame occurred. Still, it is possible that partially damaged hair cells experienced some transient recovery. The central regions of the vestibular receptors are known to contain the highest concentration of type I hair cells and calyx terminal afferents in a number of species (41). In fact, in mice, the central zone has a significantly higher (1.5:1) type I:type II hair cell ratio, than in the periphery (41). Since in mammals, little to no spontaneous hair cell regeneration occurs following loss, it is likely that the stereocilia damage observed following blast in the present findings would remain permanent (42). For reference, gentamicin-induced hair cell loss in the vestibular cristae through middle ear injections typically does not result in total type I hair cell loss (43, 44). Even though viable type I hair cells remained, animals 1 month post gentamicin administration had severe attenuation of stimulus-evoked afferent discharge modulation. However, spontaneous discharge rates were consistent throughout all testing intervals (44). Hair cell damage caused by frontal blast-wave exposure could be significant enough to cause the vestibular pathologies observed. As shown in previous studies, total hair cell loss is not required to alter vestibular sensory dysfunction.

Given the findings regarding the stereocilia loss in the vestibular receptor organs due to blast exposure, one would predict that an effect would be observed in vestibular related response behaviors. Our findings of several key behavioral measures supported that expectation. First, we observed deficits in the rotarod and righting reflex responses. Immediately following blast exposure, animals were only able to remain on a rotating rod with increasing speed for a few seconds, which was about half the time normal animals can perform the test. Their ability to remain on the rod improved after a week post-blast exposure, but was still less than normal and not until 2 weeks post-blast exposure did the animal’s ability to balance return to normal values. Likewise, animals were initially unable to right themselves after being inverted 1 day post-blast exposure for many seconds and that inability lasted up to 2 weeks after blast. However, when tested 28 days post-blast exposure, the animals could right themselves in times approaching pre-blast levels. Similar results have been observed in other blast studies performed in rodents (45, 46). Abnormalities in rotarod performance persisted for up to 1 week or longer. Rats exposed to a front 74.5 kPa blast wave had impaired performance on both the Morris water maze and balance beam test (45). Furthermore, multiple studies have observed increased righting reflex latencies in rodents after exposure to a front blast wave (45, 47). Since we observed significant hair cell stereocilia damage that remained during the entire 28-day post-blast recovery period in these animals, any improvement in vestibular related postural behavior must be due to compensation in the central vestibular circuits. Vestibular compensation has been observed in human patients and in animal studies following peripheral vestibular deficits and is likely due to alterations in central microcircuit recovery and alterations in vestibular pharmacology such as glutamate and GABA (48–52). Glutotoxicity caused impaired vestibular behaviors in rodents, such as impaired landing response and inability to right themselves in the air, with little recovery after 1 week (53). In a recent human study conducted over a period of 13-week period following unilateral peripheral vestibular loss, central compensation was observed in afflicted patients with a significant reduction in VOR asymmetry (48). Similar improvements in the VOR have been observed in rodents (54). After unilateral injury of the inner ear, glutamate receptor delta-2 subunit KO mice, a receptor selectively localized to cerebellar Purkinje cells, displayed some VOR improvement; however, this recovery time was significantly longer than wild type mice (54).

Due to damage sustained in the horizontal semicircular cristae, we expected lower hVOR gains to be observed in blast-exposed mice. However, we initially observed an increase in hVOR gains (hypermetric response) 1 day post-blast exposure. Subsequent days’ post-blast exposure exhibited gain decreases and phase lags that lasted through post-blast day 28. Hyperactive VOR gains have been reported in older patients that exhibit signs of cellular degeneration (55); however, there have not been any current reports observing this phenomenon in blast patients. In our findings, this sequelae appeared to be time sensitive and was only observable shortly after a blast-wave exposure.

There is evidence that service members with blast exposure experience significant dizziness within 72 h of injury (13). However, other studies have demonstrated that significant dizziness can persist for 6 months or longer, even up to years in blast patients (56). Similar to patient data, our mice had significantly lower VOR gains at days 14 and 28 post-blast. VOR reduced gains were actually observed at the higher frequencies of motion at day 7 post-blast, similar to previous reports (35, 36). Damage to the horizontal semicircular cristae is the first indication that there is most likely damage to the peripheral vestibular system. Due to the loss of stereocilia bundles, the signal of the innervating vestibular afferents would be predicted to be greatly reduced.

Optokinetic impairment was seen shortly after exposure to a blast wave and persisted for some time after. Some OKN recovery was observed 1 month post-blast-wave exposure. Constant rotation head velocity signals carried by canal afferents decay with a time constant of 3–5 s (57). The time constant of neural activity in the vestibular nuclei is 15–25 s which indicates there is central vestibular processing that lengthens the response time of the cupula signal, termed velocity storage (58–60). Previous studies have demonstrated that vestibular-only neurons are significant components in the velocity storage mechanism while position-vestibular-pause neurons likely help transition the vestibular signal to the oculomotor system (61). Human studies have shown a significant increase in pathological optokinetic eye movements in patients with unilateral or bilateral vestibular nucleus lesions (62). Patients with bilateral vestibular lesions had significantly reduced OKN gains (62). The medial vestibular nucleus is hypothesized to be the locus of the neural integrator for horizontal eye movements (63). Lesions of the medial vestibular nucleus cause a drastic reduction in optokinetic pursuit gains and significant reduction in the time constant of the vestibular signal. Before medial vestibular nucleus lesioning, the time constant of the vestibular signal was estimated to be 20 s, and after lesion, the time constant was significantly reduced to a minimum of 200 ms (63). The optokinetic profile of the mice after exposure to a blast-wave overpressure indicates there could be significant damage to the central vestibular system.

Given the increasing use of explosive devices in combat and civilian environments, the study of blast-related traumatic vestibular injuries has become increasingly important. The ear is especially important to study in this scenario because it serves as a pressure transducer for sound waves. Thus, the ear is typically affected first by a primary blast injury. When examining patients for evidence of ear injury, observing examining the TM is a relatively easy task that can be performed with simple tools. In fact, many human case studies examining damage to the ear following blast exposure have concentrated on TM perforation and hearing loss. However, post-blast effects to the vestibular system are common and deserve further investigation.

## Ethics Statement

This study was carried out in accordance with the recommendations of Guide for the Care and Use of Laboratory Animal. The protocol was approved by the Baylor College of Medicine Institutional Animal Care and Use Committee.

## Author Contributions

SL performed the development and analysis of the experiments and drafted the article. JD guided the development of the project and article.

## Conflict of Interest Statement

The authors declare that the research was conducted in the absence of any commercial or financial relationships that could be construed as a potential conflict of interest. The reviewer AM and handling Editor declared their shared affiliation.
